# Hydrogel Dressings for the Treatment of Burn Wounds: An Up-To-Date Overview

**DOI:** 10.3390/ma13122853

**Published:** 2020-06-25

**Authors:** Alexandra Elena Stoica, Cristina Chircov, Alexandru Mihai Grumezescu

**Affiliations:** Department of Science and Engineering of Oxide Materials and Nanomaterials, Faculty of Applied Chemistry and Materials Science, University Politehnica of Bucharest, 1-7 Gheorghe Polizu Street, 011061 Bucharest, Romania; alexandra.oprea@biointerfaceresearch.com (A.E.S.); cristina.chircov@biomedicalengineering.international (C.C.)

**Keywords:** hydrogels, burn injury, skin regeneration, wound healing, wound dressing

## Abstract

Globally, the fourth most prevalent devastating form of trauma are burn injuries. Ideal burn wound dressings are fundamental to facilitate the wound healing process and decrease pain in lower time intervals. Conventional dry dressing treatments, such as those using absorbent gauze and/or absorbent cotton, possess limited therapeutic effects and require repeated dressing changes, which further aggravate patients’ suffering. Contrariwise, hydrogels represent a promising alternative to improve healing by assuring a moisture balance at the burn site. Most studies consider hydrogels as ideal candidate materials for the synthesis of wound dressings because they exhibit a three-dimensional (3D) structure, which mimics the natural extracellular matrix (ECM) of skin in regard to the high-water amount, which assures a moist environment to the wound. There is a wide variety of polymers that have been used, either alone or blended, for the fabrication of hydrogels designed for biomedical applications focusing on treating burn injuries. The aim of this paper is to provide an up-to-date overview of hydrogels applied in burn wound dressings.

## 1. Introduction

**Figure 1:**
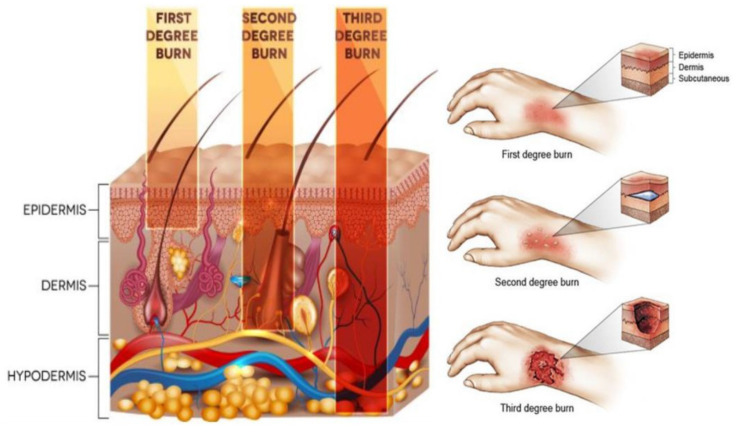
Different depth of invasion for burn injury [13]. Reprinted from an open-access source.

Without discredit, the skin is the most exposed to various impairments, such as injuries, scratches, and burns, among all human body organs. Injuries of the epithelium and connective structures are associated with a weakened ability of the human body to assure adequate protection against harms from the outer environment [1]. As stated by the World Health Organization, burn injuries represent a major public health crisis, and are among the most severe injuries with over 180,000 annual deaths worldwide [2,3,4]. Burns are defined as damages of the skin caused by excessive heat or caustic chemicals, as the most common causes [3,5,6]. Among the three types of burns, third-degree burns, also known as full-thickness burns, will destroy the entire thickness of the skin, provoking immediate cell death and matrix destruction, with the most devastating damage at the surface of the wound (see Figure 1) [7,8]. During the last years, development in acute burn management has decreased mortality rate, allowing the survival of patients with burn injuries covering up to 100% of the body surface [9,10,11,12].

**Figure 2:**
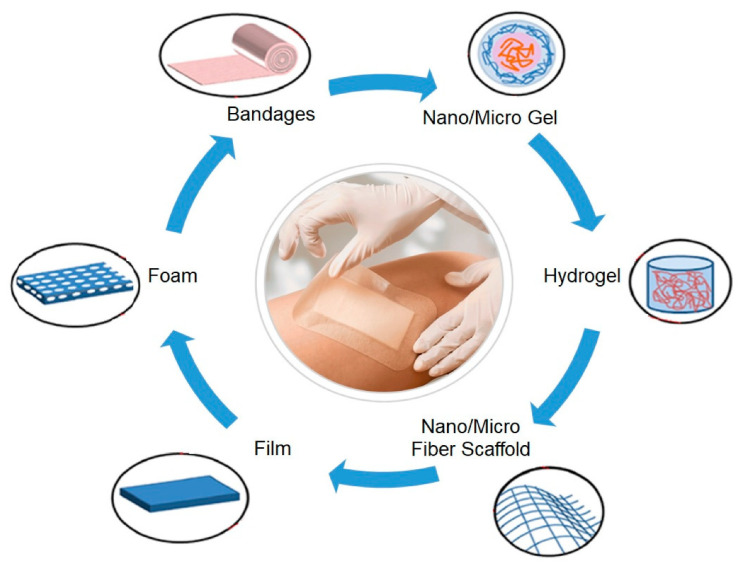
Structure of different types of dressings [24]. Reprinted from an open-access source.

Burn injuries cause disruptions of the normal skin barrier and impairments of numerous host defense mechanisms that prevent infections [14,15]. Consequently, until full epithelialization occurs, burn patients remain vulnerable to various invasive microbial infections [16]. An inappropriate repair process could induce severe damage, like the initiation of an infection or the loss of skin, which could consequently harm the subjacent tissues and even the whole organism [17,18]. The installation of infection represents one of the most usual and inevitable obstacles in the process of wound healing, especially in chronic wounds [19,20,21], and one of the most important and serious complications that could appear during the acute period subsequent to burn injury [22,23]. Although numerous dressings are already commercially available (Figure 2), there is an urgent need for the development of novel wound care treatment options to address the increasing number of burn injuries [1].

**Figure 3:**
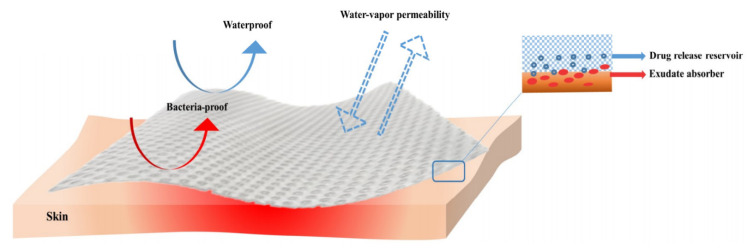
Properties of an ideal wound dressing [19]. Reprinted from an open-access source.

As previously mentioned, there is a great variety of wound dressings on the market that are used for burn wound healing. While cotton gauze is extensively used for burn care, there are some disadvantages that must be considered, namely the pain caused by its removal and possible delays in the wound healing process [22,25]. Since wound healing is a considerably dynamic process, the performance requirements of dressing should be modified as healing progress [6,26,27]. Nevertheless, a warm and moist environment has been widely accepted as the key factor that promotes fast healing, and, therefore, most modern wound care products are designed to assure these conditions [28,29,30]. Based on the “wet wound healing theory”, a wet healing environment is optimal for the growth of the granulation tissue and for facilitating skin cells division, further promoting complete wound healing [31,32,33,34]. An optimal dressing (Figure 3) is thus capable of preserving high humidity levels at the wound site whereas also erasing excess exudates; in addition, it must be non-toxic, non-allergenic, comfortable, and cost-efficient, allow for oxygen and water vapor exchange and protect against microbial invasion [19,35,36,37,38,39,40,41,42,43,44,45,46,47,48,49]. Modern wound dressings are developed as carriers for the delivery of therapeutic agents at the wound site in a variety of forms, including nanofibrous mats [50,51,52], sponges [41,53,54,55], films [30,56,57,58,59,60], foams [61,62,63,64,65], and hydrogels [1,24,66,67,68,69,70,71,72,73].

Hydrogels are generally obtained by mixing two different polymers in order to achieve a mixture with excellent wound dressing characteristics compared to the pure polymers. In this manner, hydrogels could potentially combine the characteristics of moist wound healing with an adequate fluid absorbance, while also allowing for the monitorization of the healing process owing to their transparency [28,73,74]. The intrinsic ability of hydrogels for promoting skin healing and regeneration has been an increasing studying focus, with clinical setting applications since 1980 [75].

As they are capable of satisfying important dressing requirements, wound dressings based on hydrogels are one of the most promising materials applied in wound care. Such requirements include maintaining the wound moist whereas absorbing excess exudate, covering the sensitive underlying tissue without adherence, decreasing pain through cooling effects, and actively intervening in the wound healing process [1,76,77]. However, as hydrogels cannot eliminate the pathogenic microbes by themselves, the problem associated with burn wound infections is still challenging [22]. The innovation in advanced wound care is directed to the development of active dressings, where hydrogels are combined with components that enhance the primary purpose of providing a beneficial environment for wound healing [78]. In this regard, novel strategies focus on developing hydrogels as burn wound dressings with antimicrobial properties. This paper aims to provide an up-to-date overview of the most recent strategies for developing hydrogel dressings for the treatment of burn wounds and the prevention of burn wound infections.

## 2. Inert Hydrogels for Treatment of Burn Wound Dressings

Owing to their hydrophilic character and properties similar to soft tissues, polymeric hydrogels are considered as the first biomaterials candidates in the development of wound dressings for the treatment of burn wounds [1,79,80,81]. In this context, polymeric hydrogels assure an ideal moist environment for the healing process, while also being comfortable to the patient owing to their cooling effect and non-adherent character [20,75]. More importantly, recent studies in the field of regenerative medicine demonstrate at least a partial skin regeneration in vivo through the action of bioactive hydrogels (Table 1) [75].

The wound healing process is directly affected by various local factors, oxygenation, and infection. The development of a suitable material for covering the wound and further prevent infection is a long-established requirement [82]. Researchers have been using different natural polymers, such as alginate [81,83,84,85], chitosan [81,86,87,88], collagen [89,90], dextran [91,92], hyaluronan [2,93], xanthan [94,95], konjac [95], and gelatin [96] for the production of hydrogels. Their wide application in wound dressing fabrication is based on their similitude to the extracellular matrix (ECM), which further improves acceptance by biological systems through the inhibition of the immunological reactions frequently observed for synthetic polymers [81,97].

Owing to its good elasticity and capacity to absorb a high amount of fluids, which further induces adequate moisture at the site of the wound, alginate is considered a great wound dressing material [81]. Stubbe et al. [83] developed a gelatin-alginate hydrogel for burn wound treatment. The hydrogel dressings proved good biocompatibility with adaptable cell attachment properties. Nuutila et al. [84] used a Platform Wound Device (PWD) based on alginate hydrogels embedded with high concentrations of topical antibiotics for studying the immediate treatment of burn wounds. The PWD represents a platform technology that starts as a first point treatment strategy that protects the wound and allows for the administration of topical therapeutics. The device can be adjusted to suit any size burn over any body contour. They proved a safe delivery of the antibiotics in high concentrations embedded in the alginate hydrogel using the PWD and, therefore, a successful treatment method for burn infections.

Moreover, chitosan promotes wound healing through a series of mechanisms, including fibroblasts activation, deposition, and arrangement of collagen fibers regulation, cell migration, granulation, and vascularization promotion [81]. Hence, chitosan is one of the most widely used biomaterials for hydrogel production, which is further applied for wound dressing development [87]. A hydrogel sheet (HS) composed of chitosan, honey, and gelatin was developed by Wang et al. [86] as a burn wound dressing. The histological examination showed a complete repair of the epidermis on day 12 after treatment with the HS. In addition, the toxicological evaluations demonstrated that HS is non-toxic and non-irritant material for the body and skin. Furthermore, Mingcui et al. [88] developed a porous nanocomposite hydrogel based on keratin, chitosan, and zinc oxide nanoparticles using the lyophilization technique. The results proved good tensile strength, antibacterial activity, sustained swelling and biodegradation, and excellent cell feasibility. In addition, the animal model results confirmed that the developed dressing assures about 92% repair of fractional depth injury in two weeks. In this manner, they proved that these hydrogels could be used in first-degree burn wound healing applications.

**Figure 4:**
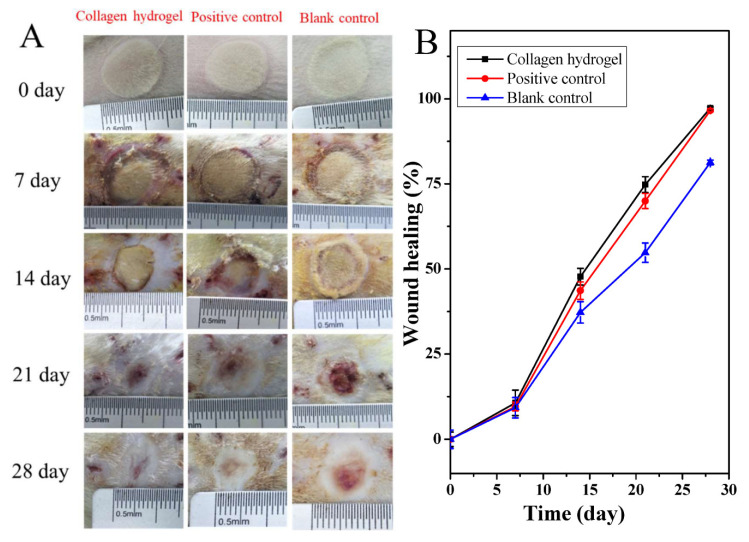
Healing of deep second-degree burn of rat skin with different treatments. (**A**): Photographs of deep second burn wounds at 0, 7, 14, 21 and 28 days. (**B**): Wound healing rate with different treatments. Positive control group, treated with commercial product (3MTM Tegaderm^TM^ hydrocolloid dressing); Collagen hydrogels group, treated with collagen hydrogel dressing containing 10 mg/mL PSC; Blank control group, without any treatment after wound burned [90]. Reprinted from an open-access source.

As collagen plays fundamental roles in ECM formation and cell and tissue development and migration, collagen-based hydrogels have been considered as potential wet dressings for wound treatment. They are highly advantageous in terms of closely fitting the wound and providing adequate moisturizing, while also preventing bacterial infections. Moreover, collagen molecules may promote wound epithelialization and accelerate wound healing [90]. Oryan et al. [89] designed a study for investigating the impact of collagen hydrogel scaffold dressing with or without the topical use of *Saccharomyces cerevisiae* on cutaneous burn wound healing in rats. The results proved increased wound healing by enhancing epithelialization and decreasing scar size, and good biomechanical properties at the wound site. Using the self-aggregating property of collagen, Ge et al. [90] prepared a novel hydrogel dressing based on a high concentration of pepsin-soluble collagen. The experiments provide clear proof and essential data for the use of aquatic origin collagen as hydrogel-based wound dressings for the treatment of refractory wounds like extensive deep burn wounds (see Figure 4).

Dextran, a polysaccharide that can potentially increase hemocompatibility of the associated materials, has numerous effects on blood coagulation homeostasis, such as diminished fibrin polymerization, platelet activation inhibition, and erythrocyte rouleaux formation [91,107]. In 2018, Zhu et al. [92] manufactured a dextran-hyaluronic acid hydrogel enriched with sanguinarine-containing gelatin microspheres. Characterized by large porosity and high swelling ratio, these systems improved fibroblast cell proliferation and sustained the release profile of sanguinarine. The results suggest that the hydrogel provides a potential high-quality strategy for the treatment and scar inhibition of infected burn wounds. A hydrogel dressing was prepared by Zheng et al. [108] using a solution blend comprised of polyvinyl alcohol and dextran-aldehyde, that was crosslinked via the freeze-thaw method and freeze-drying. Thorough evaluations revealed an excellent acceleration of the wound healing process and improved wound contraction rate and skin regeneration in a full-thickness skin defect model. Thus, the suitability of this hydrogel for application as a wound dressing has been proved.

Hyaluronic acid is a natural glycosaminoglycan that may be found in numerous human tissues, such as connective tissues, skin, synovial fluid, and umbilical cord. As a result of its biodegradability, biocompatibility, and ease of chemical modification, hyaluronic acid-based biomaterials have been largely applied in tissue engineering and for the treatment of inflammatory diseases and wounds [2,109,110,111]. Li et al. [93] developed a promising hydrogel based on hydrazide-modified hyaluronic acid and benzaldehyde-terminated F127 triblock copolymers. The obtained hydrogel combined multiple functions (i.e., adaptable mechanical strength, rapid gelation, liquid-absorption, self-heal ability, drainage, tissue adhesion, and excellent biocompatibility) in one system, proving its potential for promoting burn wound healing. Dong et al. [2] developed an improved method of adipose-derived stem cells (ASCs) delivery for the treatment of burn wounds. Specifically, the method used an in situ-formed hydrogel system consisting of hyperbranched polyethylene glycol diacrylate polymer, a commercially available thiol-functionalized hyaluronic acid, and a short RGD peptide. The developed hydrogels provided an effective niche that could enhance the regenerative potential of ASCs and promote burn wound healing.

Xanthan gum is a high molecular weight anionic heteropolysaccharide. Its backbone consists of (1,4)-β-D-glucose residues with a trisaccharide side chain linked at the C3 position to alternate glucose residues [94,112]. Xanthan gum is a microbial and exo-polysaccharide, which has been utilized in biomedical applications owing to its great biocompatibility and gelling properties [113,114]. Shawan et al. [94] fabricated xanthan gum and gelatin hybrid composite hydrogels for evaluating its skin wound healing efficiency using experimental skin burn wounds in rats. The results proved good polymeric networks, with adequate porosity of the hydrogels, biodegradability, and good wound healing ability.

Therefore, it can be observed that natural polymers are biomaterials of significant importance for wound dressings development, as they promote wound repair and healing processes through a variety of physiological mechanisms. Moreover, their efficiency is also based on their intrinsic antimicrobial characteristics, which prevent wound infections.

## 3. Active Hydrogels for Treatment of Burn Wound Dressings

Although a moist environment is required at the wound site, it may also increase the risk of microbial infections, which will further extend the wound and/or affect the wound healing process [115]. Microbial colonization is not desired, as it may conduct serious infections, which can result in disease, disability, or even death [116]. The natural reparative and regenerative phases implicated in the healing process fail to occur when wounds are colonized by opportunistic microbes [117]. Additionally, uncontrolled infections may impede the regeneration of the anatomical and physiological structures and culminate in chronic non-healing wounds [70]. To prevent and combat infections, advanced medicine relies on antimicrobial agents like antibiotics, which act by either destroying pathogens or inhibiting their growth [118]. Hence, hydrogels with antibacterial characteristics have great potential in clinical applications [119]. Unfortunately, the wrong use of antibiotics has led to the development of increasingly multi-resistant microbes [118]. The rise of multi-resistant bacterial and fungal infections in burn wounds has increased the need for novel burn wound treatment strategies’ development [120]. Generally, there are two categories of antimicrobial agents, namely organic agents, including antibiotics and organic mineral salts, and inorganic agents, including silver [120,121,122], zinc [88,119], and copper [119,123]. In recent years, antimicrobial agents-embedded wound dressings have appeared as a viable alternative to decrease wound microbial colonization and infection for improving the healing process [19].

### 3.1. Active Hydrogels Based on Quaternary Ammonium Salts for Wound Dressings

In this context, Gharibi et al. [91] developed quaternary ammonium salts (QAS)-containing wound dressing membrane and utilized dextran to counterbalance the adverse effects of the antimicrobial agent. Despite the high antimicrobial efficiency of quaternary ammonium salts, their hostile hemolytic effect on red blood cells is a challenging problem for using them as an active antiseptic agent in wound dressings. Wound dressings were prepared using the sol-gel hydrolysis and polycondensation reaction of a methoxysilane-functionalized quaternary ammonium compound and a methoxysilane-terminated polyurethane prepolymer, at various concentrations, and subsequently surface-modified with dextran. The antimicrobial activity of the dextran-grafted samples was maintained, while also proving potential hemocompatibility and good cytocompatibility in fibroblast cell cultures.

Additionally, Li et al. [124] evaluated the potential of maleopimaric acid quaternary ammonium cation (MPA-N^+^) based on rosin acid as a bactericide for modified cotton textiles (CT) considering the fact that antimicrobial CT show great promise for wound dressings. Obtained results confirmed that MPA-N^+^ modified CT (CT-g-MPA-N^+^) can be applied for wound dressings and CT modification using MPA-N^+^ demonstrates a new strategy for using renewable resources to control the spread of infectious diseases.

Furthermore, Zhou et al. [125] prepared an eco-friendly dressing using a chitin-derived membrane with amphipathic anion/quaternary ammonium salt designed for antibacterial purposes. Successfully prepared chitin-amphiphilic ion/quaternary ammonium salt dressing present antibacterial and antipollution effects and promote wound healing. Overall, this study reveals a promising new material for a natural dressing for wound application.

### 3.2. Active Hydrogels Based on Silver for Wound Dressings

Many silver-based products have become effective substitute agents in burn management in order to avoid the use of antibiotics, with an increased number of studies stating their effectiveness against a large range of microbes [126,127,128,129]. The important antimicrobial activity of silver nanoparticles has been previously reported [120,121,122,130]. As silver treatments applied in burn wound care have also been associated with toxicity for human cells [120], the balance between cytotoxicity and antimicrobial activity of wound dressings must be considered when applied at the wound site [120,130]. Nonetheless, the increase of antibiotic-resistant bacteria has forced to re-evaluate the character of silver and silver derivatives as antibacterial agents for restraining the colonization of bacteria in burn injuries [131]. Boonkaew et al. [120] compared the antimicrobial efficiency of a novel silver hydrogel dressing with two commercially available silver wound dressings for burns, namely Acticoat™ and PolyMem Silver^®^. They proved that after 24-h exposure, the silver hydrogel decreased most of the tested microbial strains below the detection limit and reduced the viability of bacteria by 94–99%. Furthermore, a thermo-sensitive hydrogel consisting of methylcellulose and embedded silver oxide nanoparticles was prepared by Kim et al. [121] through the one-pot synthesis method in which a silver acetate precursor salt induces a salt-out effect in the methylcellulose solution. They proved that the obtained thermo-responsive methylcellulose hydrogel has important potential for wound regeneration, considering its great antimicrobial and burn wound healing activity. In a study performed by Banerjee et al. [122], a novel treatment for promoting vascularization in burn wounds was proposed. They developed a two-step treatment method based on the controlled time and dose release of silver sulfadiazine and the subsequent delivery of ASCs, which aids in preventing silver toxicity related to traditional topical delivery methods and stimulates the regeneration of the wound. A PEGylated fibrin hydrogel containing 50 mg of silver sulfadiazine-loaded chitosan microspheres was applied on the wounds and results demonstrated that the proposed sequential treatment for infected burn wounds reduces bacterial infection, while also promoting neo-vascularization and improved matrix remodeling.

The hydrogels based on 2-hydroxyethyl acrylate and itaconic acid were synthesized by Vuković et al. [132] and used for silver(I) ions incorporation. The obtained hydrogels presented promising antibacterial activity against methicillin sensitive *S. aureus* (MSSA) and methicillin resistant *S. aureus* (MRSA), indicating the capacity to treat the life-threatening infections.

Therefore, it can be observed that silver nanoparticles are widely applied in wound dressings due to their low toxicity for human cells, naturally availability, and strong antimicrobial effects.

### 3.3. Active Hydrogels Based on Zinc for Wound Dressings

Zinc is a highly necessary element for the human body, and, owing to its complex antibacterial mechanisms, it is significantly efficient on various antibiotic-resistant strains [119]. Zinc oxide nanoparticles possess bactericidal character, and it is currently applied as a part of a large variety of restorative materials [133,134]. Mingcui et al. [88] fabricated a nanocomposite hydrogel consisting of keratin, chitosan, and zinc oxide nanoparticles as an antimicrobial strategy for burn wound healing. The mechanical properties, swelling ability, bactericidal effect, and biocompatibility of the nanocomposite were evaluated for its effectiveness for burn wound treatment. Khorasani et al. [135] incorporated zinc oxide nanoparticles into heparinized polyvinyl alcohol/chitosan hydrogels for wound dressing applications. Based on the results, the obtained bionanocomposite hydrogels improved the performance in the wound healing process as it efficiently protected the surface of the wound against exudate accumulation and dehydration, while impeding bacteria growth and infection development.

Furthermore, Rakhshaeia and Namazi [136] prepared flexible nanocomposite hydrogel films through combination of zinc oxide impregnated mesoporous silica (ZnO-MCM-41) as a nano drug carrier with carboxymethyl cellulose (CMC) hydrogel. The antimicrobial property of the obtained CMC/ZnO-MCM-41 samples is a result of intrinsic antibacterial activity of ZnO nanoparticles and confirmed the prolonged release of TC. The authors affirm that the obtained hydrogels could serve as a kind of promising wound dressing with sustained drug delivery properties.

Additionally, Khorasani et al. [137] prepared polyvinyl (alcohol)/chitosan/nano zinc oxide nanocomposite hydrogels using the freeze-thaw method. The results of toxicity and antibacterial activity of samples indicated that obtained hydrogels were non-toxic and biocompatible and were significantly capable to protect the wounds against microorganisms.

### 3.4. Active Hydrogels Based on Growth Factors, Cytokines, and Cells for Wound Dressings

Regardless of the great number of active compounds that could be considered as therapeutics for promoting wound healing, the wound inflammatory environment inhibits their activity to improve healing, with a limited number of candidates proving clinical effects [1,2]. Analgesics such as morphine [138], ibuprofen [139,140], or lidocaine [141,142] are of significant interest in extensive burns, infected wounds, or in palliative medicine [1,143]. Additionally, hydrogels may also deliver growth factors [1,144,145], stem cells [2,146], peptides [147,148,149], and various drugs, such as anti-inflammatory drugs [144,150,151], amino acids [152], antioxidants [70,153], vitamins [154,155] and nutrients [135,156], which may decrease the inflammatory reaction, nourish the wound tissue, and promote wound healing [119,126]. One of the recent classes of bioactive hydrogel wound dressings is based on the healing properties provided by growth factors [1,157], cytokines [158], or cells [1,158]. This section also contains recently published literature studying the effects of applying natural alternatives, such as honey, bacterial cellulose, or aloe vera as regenerative and antibacterial agents that further accelerate wound healing processes.

Nimal et al. [126] prepared an injectable hydrogel comprising nano tigecycline and chitosan platelet-rich plasma with an anti-staphylococcal activity using *Drosophila melanogaster* model for infectious wounds. This hydrogel provided an appropriate medium for the delivery of antibiotics and effectively prevented skin infections.

Furthermore, Wang et al. [144] produced a hydrogel consisting of chemically modified hyaluronic acid, dextran, and β-cyclodextrin and integrating resveratrol and vascular endothelial growth factor (VEGF) plasmid, which acts as an anti-inflammatory and pro-angiogenic components for burn wounds. The hydrogel scaffold was loaded with plasmid DNA encoded with VEGF and conjugated with polyethyleneimine. Wounds treated for 21 days with these hydrogels demonstrated enhanced wound healing by inhibiting inflammation and promoting angiogenesis compared to untreated wounds and hydrogel-alone treated wounds, suggesting that the in situ formed hydrogels may be applied for wound healing and tissue regeneration applications. Moreover, Mohamad et al. [158] performed an in vivo evaluation of bacterial cellulose and acrylic acid wound dressing hydrogels containing keratinocytes (HEK—human epidermal keratinocytes) and fibroblasts (HDF—human dermal fibroblasts) for burn wounds. Wound healing was accelerated in mice treated with hydrogels and hydrogels embedded with cells healed quicker, by contrast to when no treatment was administered, with best results associated with the delivery of HEK and HDF cells. Therefore, the prepared hydrogels can act as potential materials and cell carriers for the rapid healing of burn wounds.

Based on their broad-spectrum antimicrobial activity and reduced probability of inducing drug resistance, antimicrobial peptides are a new generation of potential antimicrobial molecules [148]. Zhou et al. [147] investigated a bioactive peptide amphiphile nanofiber-based hydrogel biomaterial that may stimulate burn wound healing. Burn wounds in rats were treated with the bioactive Arg-Gly-Asp-Ser (RGDS)-modified gel that proved important cell proliferation in vitro. The in vivo assays showed that the RGDS- peptide amphiphile gel notably improved the burn wound healing process between day 7 to 28 through enhanced re-epithelialization. Application of these gels accelerates deep partial-thickness burn wound recovery by stimulating fibroblasts and creating a suitable environment for the proliferation of epithelial cells and closure of the wound.

Additionally, Khan et al. [148] developed a hydrogel that has the potential for treating bacterial wound infections. The hydrogel formulation is based on an antimicrobial peptide, ε-poly-l-lysine, and catechol, which was cross-linked via mussel-inspired chemistry between the amine and phenol groups. In addition to its antimicrobial properties, they demonstrated that the hydrogel presents antibiofilm activity toward multidrug-resistant bacteria. In addition, in vivo studies indicated a considerable reduction in more than four orders of magnitude of the bacterial burden in the infected burn wounds. As it is biocompatible and noncytotoxic to mammalian cells, this hydrogel could be applied in burn wound care.

Since they are appropriate carriers for low soluble drugs or bioactive molecules, liposomes are able to overcome this hydrogel limitation. Hence, by combining these two delivery systems, an encouraging alternative to reach controlled dermal drug delivery, and effective localized skin therapy could be developed [159]. Wu et al. [160] studied the liposome-encapsulated farnesol in order to improve tissue repair in rat models of third-degree burns. The wounds were treated for 1 and 2 weeks with a formulated gel comprising different ratios of 2% hydroxypropyl methylcellulose and 4 mM liposomal farnesol. The liposomal gels prepared in this study enhanced collagen production and wound healing both in vitro and in vivo, but inhibited fibroblast proliferation at high concentrations. The gels exhibited notable effects on wound healing of third-degree burns compared with the untreated or the hydroxypropyl methylcellulose gel alone and commercial silver sulfadiazine cream treated groups. Moreover, the capacity of liposomes to provide sustained drug/substance release could allow for targeted drug delivery to specific skin layers. The rapid liposome clearance from the skin site may be prevented through the use of this hydrogel, which ensures additional protection against fast degradation by conserving the liposomal membrane integrity. The characteristics of the hydrogel in terms of mesh size, porosity, and polymer composition and the physicochemical properties of the liposomes, such as size, composition, and surface charge, directly determine the release of drug/substance [159].

### 3.5. Active Hydrogels Based on Natural Agents for Wound Dressings

As antibiotics are progressively becoming resistant by infection-producing strains, researchers are currently focusing on the large bioresource repertoire. They mainly consist of herbs but can also include animal and mineral ingredients [19,161,162,163,164]. There are a lot of natural agents with bioactive effects on wounds with complications from polymicrobial infections mentioned in the literature. At the biofilm level, the bactericidal effects of such agents target both the initial and the advanced phases of wound infections [19,164].

**Figure 5:**
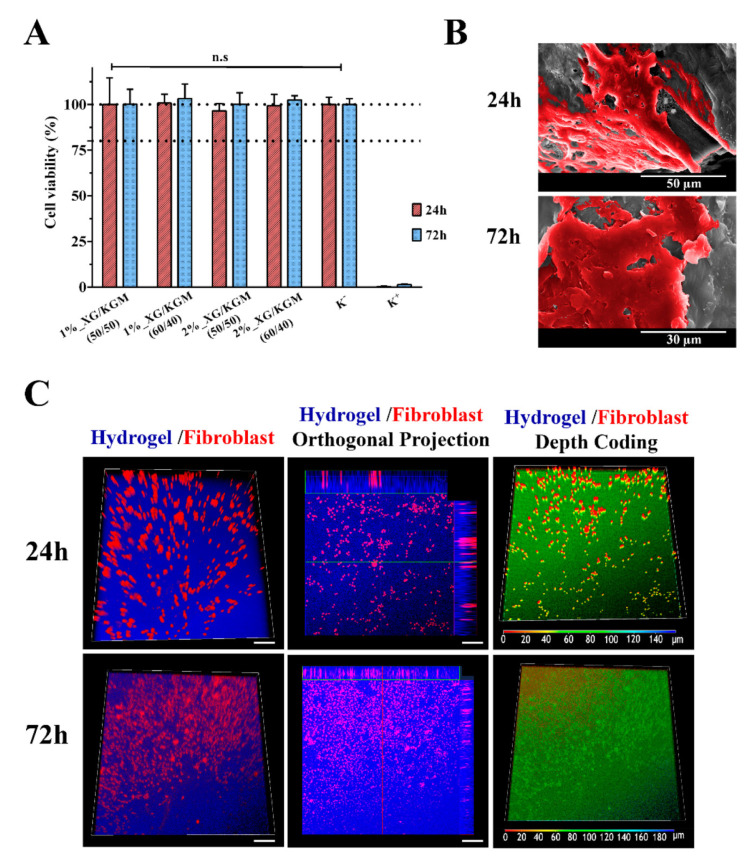
Characterization of the cytocompatibility of the hydrogels. (**A**) MTT assay of human fibroblast cells grown in the presence of different hydrogels. Wells treated with ethanol were used as positive controls. n.s: no statistically significant groups. The data are shown as means ± standard deviations (*n* = 3). (**B**) Representative SEM images of fibroblast cell adhesion and proliferation on the surface of the 1%_XG/KGM_(60/40) hydrogel, after 24 h and 72 h of incubation. (**C**) Confocal laser scanning microscopy (CLSM) images of cell internalization in 1%_XG/KGM_(60/40) after 24 h and 72 h, where different colors correspond to distinct depth values (as indicated in the color-coding scale) [95]. Reprinted from an open-access source.

**Figure 6:**
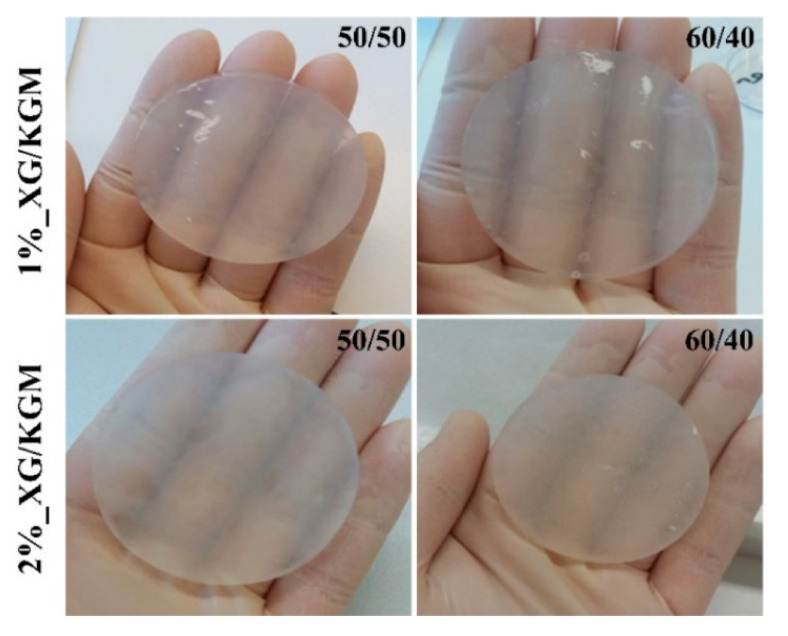
Macroscopic images of the hydrogels XG/KGM [95]. Reprinted from an open-access source.

Not long ago, naturally-occurring materials gained renewed attention for biomaterial applications due to their important biocompatibility, antimicrobial, and environmentally friendly properties [19,81,165]. Konjac glucomannan, a plant derivative from the Amorphophallus konjac corm, a native plant from China and Japan, is an example of such natural biomaterials [41,53,166,167,168]. Zhou et al. [168] fabricated matrine-loaded composite hydrogel consisting of Konjac glucomannan and fish gelatin as an antimicrobial wound dressing. The bioactive compound improved the antibacterial activity of the gels by maintaining the physiological environment for wound healing and preventing bacteria growth on the surface of the wound. Alves et al. [95] obtained a thermo-reversible hydrogel comprising of xanthan gum and konjac glucomannan (Figure 5) at different concentrations and ratios. The obtained hydrogels showed a transparent and moisturized appearance (Figure 6), which permitted the continuous observation of the wound healing process without dressing removal. The obtained hydrogels are hydrophilic, thus providing a moist environment, while also absorbing the excess exudate and suitable biological properties for promoting cell adhesion, migration, and proliferation [95].

In addition, the therapeutic features of honey in regard to wound healing applications, such as ensuring a topical nutrition to the wound, stimulating granulation, angiogenesis, and wound epithelialization, and reducing inflammation, are the main criteria that make it suitable for introduction into wound dressings [19,169,170,171]. Zohdi et al. [172] developed a crosslinked Malaysian honey-incorporated hydrogel dressing, which exhibited excellent physical properties, such as proper transparency, exudate absorbance, and acidic pH values, as ideal characteristics of burn wound dressings.

While bacterial cellulose is chemically identical with plant cellulose, the degree of polymerization for bacterial cellulose is approximately 2000–6000 and for plant cellulose, approximately 13,000–14,000 [81]. Bacterial cellulose has great hydrophilicity, water-uptake capacity, permeability, and tensile strength, characteristics that have attracted great interest as wound dressing material [81]. Moreover, bacterial cellulose is currently considered a promising functional biomaterial with various applications in different fields, including skin tissue repair, scaffolds for tissue engineering, and wound healing applications [151,158,173,174]. Loh et al. [173] performed an in vivo evaluation of a keratinocytes and fibroblasts-containing wound dressing hydrogel composed of bacterial cellulose and acrylic acid for burn wounds. They demonstrated that these hydrogels are promising for burn wound dressing and cell carrier applications.

Aloe vera has a healing action that occurs due to the maintenance of the moisture of the wound, reduced inflammatory process, enhanced cell migration and proliferation, and maturation of collagen. Its effects appear by the synergistic action among the different active components that act on the tissue during the novel epithelium formation [146,175,176]. Yates et al. [177] patented an antimicrobial therapeutic hydrogel composition comprised of a pharmaceutical and/or medical-grade silver salt, and an aloe vera gel or extract. Additionally, it may also include a non-ionic surfactant, stabilizing agents, and polyol and hydrophilic hygroscopic polymers. The so-obtained hydrogel has potential antimicrobial activity against bacteria, fungi, viruses, and protozoa, acting as an efficient treatment for burns, and as a wound/lesion dressing, that maintains adequate moisture levels and provides a physiologic environment that stimulates wound healing and pain relief. Oryan et al. [146] evaluated the in vivo effects of allogeneic ASCs-loaded aloe vera hydrogel on a rat burn wound model. They proved that aloe vera enhanced the anti-inflammatory effect of ASCs by decreasing the TGF-ß1 and bFGF expression level, diminishing scar formation. Combining ASCs with aloe vera hydrogels could bring advancements in the field of regenerative medicine as it promotes the pro-angiogenic effect of ASCs, increases the expression level of cytokines and growth factors, and improves wound repair and regeneration.

Amniotic membrane, the innermost lining of the human placenta, is a globally accepted biomaterial for the treatment of second and third-degree burns, as it contains numerous proteins, growth factors, and stem cells that enhance the wound healing process [178,179,180,181,182,183,184]. A study by Hossain et al. [184] proved the efficiency of amniotic membrane and *Moringa oleifera* are decisive agents for higher epithelialization, quicker wound healing, no rejection phenomena, decreasing number of suffering patients, and cost-efficiency. Another study was realized by Rahman et al. [178], which prepared a gel by combining amnion and aloe vera extract, which showed promising effects in internal epithelialization and diminished scar formation.

While currently available therapeutic agents are generally inadequate in regard to their efficacy and numerous adverse severe effects, natural biocompounds have been applied in medicine since ancient times as they are well known for their capabilities of promoting wound healing and preventing infection without causing significant side effects. Therefore, this class of materials should be an alternative strategy for the development of hydrogels used for the treatment of burn wounds.

## 4. Conclusions

Among the wound dressings developed, hydrogels have gained the consideration of researchers as a result of their intrinsic ability to mimic the 3D structure of the skin ECM. Moreover, hydrogels are hydrophilic 3D networks, which can absorb optimal quantities of biological fluids (e.g., wound exudate) or water. Additionally, hydrogels are capable of maintaining dry, sloughy, or necrotic wounds clean by rehydrating dead tissues (moist healing), thus leading to an increase of autolytic debridement and surface wound cooling. Consequently, hydrogels could aid in pain relief and, thus, improve patient acceptance of the dressing. Further, hydrogel biodegradability eliminates possible complications related to wound dressing replacement, like tissue maceration, infection, and pain. Despite the numerous hydrogel-based products already available on the market, advanced hydrogel dressings development or optimization still represents an important research area, with the purpose of further improving skin healing in reports to specific clinical applications. Antimicrobial hydrogels represent an important class of macromolecular antimicrobial agents, which have proved significant efficiency in preventing and treating drug-resistant infections.

## Figures and Tables

**Table 1:** The properties of the burn devices under inert hydrogels.

Polymeric Hydrogel Dressing	System Description	Tensile Strength	Antibacterial Activity	Swelling and Biodegradation	Degree Burn	Healing Time	Healing Process	Reference
**Alginate**	Alginate hydrogel/ZnO NPs	-	*Against E. coli*, *S. aureus*, *C. albicans*, *and methicillin resistant S. aureus*	16–20 swelling ratio; biodegradation in PBS (up to 40% in 3 weeks);	3rd degree	48 h	Hemostatic potential evaluated through blood clotting ability; Ex-vivo epithelialization shown through keratinocyte cells proliferation and migration towards the wounded area; effect favoured by release of Zn^2+^	[98]
	Gentamicin loaded Mannuronic alginate/amidated pectin blend microparticle	-	*Against S. aureus and P. aeruginosa*	11.91 ± 0.87–14.81 ± 0.96 swelling ratio	3rd degree	-	Optimal healing environment assured through good poweder flowability, high fluid absorbing capacity and water permeability at equiibrum	[99]
	Photocrosslinkable functionalized gelatine-alginate hydrogels	6–12 kPa storage modulus	-	More than 1200% swelling ratio	-	-	Good biocompatibility with adaptable cell attachment properties for HFF-1 foreskin fibroblast cells	[83,100]
**Chitosan**	Keratin-chitosan/ZnO NPs	0.31 MPa	Against *S. aureus and E. coli*	Up to 30 swelling ratio in 7 days; Biodegradation up to 64% in 7 days	1st degree	7–14 days	Migration of keratinocytes in epidermis	[88]
	Crosslinked carboxymethyl chitosan-dialdehyde-modified cellulose nanocrystal	4 kPa maximum storage modulus	-	Up to 350% swelling ratio	Deep partial thickness skin burn	14 days	Biocompatibility for normal adult human primary dermal fibroblasts in vitro in 2D and 3D cell models; healing at 14 days in deep partial thickness skin burn in vivo model; formation of hair follicles and blood vessels; densely packed collagen fibers with regular arrangement	[101]
	IGF-1C chitosan hydrogel							
**Collagen**	Collagen hydrogel/*Saccharomyces cerevisiae* probiotic	Ultimate tensile load approx. 60 N	-	Complete biodegradation at 20 days		22 days	Improved the wound closure, cosmetic appearance and decreased scarring at 12 and 22 days post injury (DPI); Epidermal proliferaton at 12 DPI, lower inflammation and granulation tissue formation, complete re-epithelialization at 22 DPI; normal appearance of the skin	[89]
	Acid soluble collagen/pepsin soluble collagen	-	-	-	2nd degree	28 days	Formation of new epidermis on day 14; apparition of hair follicles, sebaceous glands, dermal papillae and maturation of skin appendages on day 21; ordered fibrous tissue and high formation of skin appendages on day 28	[90]
	Collagen I-hyaluronic acid hydrogel	-	Against *S. aureus and E. coli*	Up to 95% swelling ratio at day 3; 59% maximum degradation at 7 days in enzymatic medium and 30% in enzyme free medium	-	14 days	Proliferative activity of HMEC human microvascular endothelial cells and COS-7 fibroblasts cultured within the hydrogel; increase of vascular endothelial growth factor level in HMEC; weak inflammatory behaviour at 4 days after in vivo implantation; no systemic toxicity; complete in vivo wound healing after 14 days; complete normal structure of the epithelial tissue and less inflammatory response	[102]
**Dextran**	Dextran- hyaluronic acid hydrogel-sanguinarine/gelatin microspheres	Tensile load 15N in dry state and respectively 35N in wet state	Against *methicillin- resistant S. aureus and E. coli*	Swelling ratio 29 (in water) and 25 (in PBS); Biodegradation in PBS 31% and in hyaluronidase 24% (at day 21)	-	More than 20 days	Enhancement of NIH-3T3 fibroblast cell proliferation in vitro; improvement of re-epithelialization and enhancement of extracellular matrix remodelling in rat full-thickness burn infection models; efficient scar inhibition	[92]
	Dextran hydrogels	-	-	Biodegradation promoted by early inflammatory cell infiltration	3rd degree	3–5 weeks	Early inflammatory cell infiltration; Endothelial cell penetration at day 7; mature epithelium, presence of hair follicles and sebaceous glands at day 21; new hair growth and normal epidermal morphology at 5 weeks	[103]
	Dextran/bacterial cellulose hydrogel	Up to 16 ± 2.3 MPa	-	96.7 ± 0.49% water content	-	14 days	In vitro biocompatibility for fibroblast cells; complete wound healing at 14 days; significant skin maturation, mature epithelial layer and formation of hair follicles	[104]
**Hyaluronan**	Hyaluronic acid-benzaldehyde terminated F127 triblock copolymer	Adaptable mechanical strength	-	2600–4500% swelling ratio in 3–5 min	Deep partial-thickness burn model	21 days	Moderate tissue adhesiveness; good exudation-absorption; good compatibility for 3T3 fibroblast cells; increased wound close rate with time; more typical epidermis and skin appendages compared to controls at day 21; complete epidermal wound healing at day 14	[93]
	Hyaluronic acid-poloxamer hydrogel	-	Against *E. coli* migration	-	-	14 days	Complete wound healing in rat models by day 14; promotion of fibroblast cells accumulation and collagen deposition, granulation tissue formation, angiogenesis	[105]
	Aminoethyl methacrylate hyaluronic acid-methacrylated methoxy polyethylene glycol hydrogel/chlorhexidine diacetate-nanogel	-	*Against E. coli and S. aureus*	Up to 2657.24% swelling ratio after 24 h		14 days	Rapid homeostasis; accelerated healing process	[106]

## References

[1] Koehler J., Brandl F.P., Goepferich A.M. (2018). Hydrogel wound dressings for bioactive treatment of acute and chronic wounds. Eur. Polym. J..

[2] Dong Y., Cui M., Qu J., Wang X., Kwon S.H., Barrera J., Elvassore N., Gurtner G.C. (2020). Conformable hyaluronic acid hydrogel delivers adipose-derived stem cells and promotes regeneration of burn injury. Acta Biomater..

[3] Jeschke M.G., Gauglitz G.G., Jeschke M.G., Kamolz L.-P., Sjöberg F., Wolf S.E. (2020). Pathophysiology of Burn Injuries. Handbook of Burns Volume 1.

[4] Kaddoura I., Abu-Sittah G., Ibrahim A., Karamanoukian R., Papazian N. (2017). Burn injury: Review of pathophysiology and therapeutic modalities in major burns. Ann. Burns Fire Disasters.

[5] Brassolatti P., de Andrade A.L.M., Bossini P.S., Otterço A.N., Parizotto N.A. (2018). Evaluation of the low-level laser therapy application parameters for skin burn treatment in experimental model: A systematic review. Lasers Med. Sci..

[6] Butko Y., Tkachova O., Ulanova V., Şahin Y.M., Levashova O., Tishakova T. (2019). Immune histochemical study of KI-67 level and ribonucleic acid in the process of healing of burn wounds after treatment with drugs containing dexpanthenol and ceramide. Biointerface Res. Appl. Chem..

[7] DeSanti L. (2005). Pathophysiology and current management of burn injury. Adv. Skin Wound Care.

[8] Nelson T. (2018). The Evidence for the Effectiveness of Nonpharmacological Intervention Strategies Administered During Physiotherapy for Reducing Pain in Patients who Have Suffered Burn Injuries: A Systematic Review. Master’s Thesis.

[9] Finnerty C.C., Jeschke M.G., Branski L.K., Barret J.P., Dziewulski P., Herndon D.N. (2016). Hypertrophic scarring: The greatest unmet challenge after burn injury. Lancet.

[10] McCulloh C., Nordin A., Talbot L.J., Shi J., Fabia R., Thakkar R.K. (2018). Accuracy of prehospital care providers in determining total body surface area burned in severe pediatric thermal injury. J. Burn Care Res..

[11] Chan L.-C., Lee M.-S., Ou Y.-N., Cheng H.-L., Wang C.-H. (2018). Energy requirements for ICU burn patients in whom the total body surface area affected exceeds 50 percent: A practical equation. Asia Pac. J. Clin. Nutr..

[12] Sheckter C.C., Li A., Pridgen B., Trickey A.W., Karanas Y., Curtin C. (2019). The impact of skin allograft on inpatient outcomes in the treatment of major burns 20–50% total body surface area—A propensity score matched analysis using the nationwide inpatient sample. Burns.

[13] Owda A.Y., Salmon N., Shylo S., Owda M. (2019). Assessment of Bandaged Burn Wounds Using Porcine Skin and Millimetric Radiometry. Sensors.

[14] Jahromi M.A.M., Zangabad P.S., Basri S.M.M., Zangabad K.S., Ghamarypour A., Aref A.R., Karimi M., Hamblin M.R. (2018). Nanomedicine and advanced technologies for burns: Preventing infection and facilitating wound healing. Adv. Drug Deliv. Rev..

[15] Gayas M.A., Ahmad R.A., Gugjoo M.B., Handoo N. (2018). Fungal wound infections: Mini review. Pharma Innov..

[16] Parikh D., Fink T., Rajasekharan K., Sachinvala N., Sawhney A., Calamari T., Parikh A.D. (2005). Antimicrobial silver/sodium carboxymethyl cotton dressings for burn wounds. Text. Res. J..

[17] Andrews E. (2018). Dressings in burn wound management. Pharm. Mag..

[18] Aljghami M.E., Saboor S., Amini-Nik S. (2019). Emerging innovative wound dressings. Ann. Biomed. Eng..

[19] Negut I., Grumezescu V., Grumezescu A.M. (2018). Treatment Strategies for Infected Wounds. Molecules.

[20] Denzinger M., Held M., Scheffler H., Haag H., Nussler A.K., Wendel H.P., Schlensak C., Daigeler A., Krajewski S. (2019). Hemocompatibility of different burn wound dressings. Wound Repair Regen..

[21] Mohebali A., Abdouss M., Taromi F.A. (2020). Fabrication of biocompatible antibacterial nanowafers based on HNT/PVA nanocomposites loaded with minocycline for burn wound dressing. Mater. Sci. Eng. C.

[22] Kaur P., Gondil V.S., Chhibber S. (2019). A novel wound dressing consisting of PVA-SA hybrid hydrogel membrane for topical delivery of bacteriophages and antibiotics. Int. J. Pharm..

[23] Wang Y., Beekman J., Hew J., Jackson S., Issler-Fisher A.C., Parungao R., Lajevardi S.S., Li Z., Maitz P.K. (2018). Burn injury: Challenges and advances in burn wound healing, infection, pain and scarring. Adv. Drug Deliv. Rev..

[24] Teixeira A.M., Paiva C.M., Amorim T.P.M., Felgueiras P.H. (2020). Electrospun nanocomposites containing cellulose and its derivatives modified with specialized biomolecules for an enhanced wound healing. Nanomaterials.

[25] Perchyonok V.T. (2018). Copazan herbal gel and wound healing in vitro: Assessment of the functional biomaterial for veterinary application. Biointerface Res. Appl. Chem..

[26] Sorg H., Tilkorn D.J., Mirastschijski U., Hauser J., Kraemer R. (2018). Panta rhei: Neovascularization, angiogenesis and nutritive perfusion in wound healing. Eur. Surg. Res..

[27] Rosińczuk J., Taradaj J., Dymarek R., Sopel M. (2016). Mechanoregulation of wound healing and skin homeostasis. BioMed Res. Int..

[28] Balakrishnan B., Mohanty M., Umashankar P.R., Jayakrishnan A. (2005). Evaluation of an in situ forming hydrogel wound dressing based on oxidized alginate and gelatin. Biomaterials.

[29] Hu S., Bi S., Yan D., Zhou Z., Sun G., Cheng X., Chen X. (2018). Preparation of composite hydroxybutyl chitosan sponge and its role in promoting wound healing. Carbohydr. Polym..

[30] Tan S.T., Winarto N., Dosan R., Aisyah P.B. (2019). The benefits of occlusive dressings in wound healing. Open Dermatol. J..

[31] Rowan M.P., Cancio L.C., Elster E.A., Burmeister D.M., Rose L.F., Natesan S., Chan R.K., Christy R.J., Chung K.K. (2015). Burn wound healing and treatment: Review and advancements. Crit Care..

[32] Zhao W.Y., Fang Q.Q., Wang X.F., Wang X.W., Zhang T., Shi B.H., Zheng B., Zhang D.D., Hu Y.Y., Ma L. (2020). Chitosan-calcium alginate dressing promotes wound healing: A preliminary study. Wound Repair Regen..

[33] Wang N., Yu K.-K., Shan Y.-M., Li K., Tian J., Yu X.-Q., Wei X. (2020). HClO/ClO^−^—indicative interpenetrating polymer network hydrogels as intelligent bioactive materials for wound healing. ACS Appl. Biol. Mater..

[34] Amanzadi B., Mirzaei E., Hassanzadeh G., Mahdaviani P., Boroumand S., Abdollahi M., Hosseinabdolghaffari A., Majidi R.F. (2019). Chitosan-based layered nanofibers loaded with herbal extract as wound-dressing materials on wound model studies. Biointerface Res. Appl. Chem..

[35] Nischwitz S.P., Hofmann E., Kamolz L.-P. (2019). The ideal wound dressing-beyond the ideal: A short comment on’Properties of an ideal burn dressing: A survey of burn survivors and front-line burn healthcare providers’ by T. Carta, JP Gawaziuk et al.. Burns.

[36] Ehterami A., Salehi M., Farzamfar S., Vaez A., Samadian H., Sahrapeyma H., Mirzaii M., Ghorbani S., Goodarzi A. (2018). In vitro and in vivo study of PCL/COLL wound dressing loaded with insulin-chitosan nanoparticles on cutaneous wound healing in rats model. Int. J. Biol. Macromol..

[37] Aragón J., Costa C., Coelhoso I., Mendoza G., Aguiar-Ricardo A., Irusta S. (2019). Electrospun asymmetric membranes for wound dressing applications. Mater. Sci. Eng. C.

[38] Kim Y., Doh S.J., Lee G.D., Kim C., Im J.N. (2019). Composite nonwovens based on carboxymethyl cellulose for wound dressing materials. Fibers Polym..

[39] İnal M., Mülazımoğlu G. (2019). Production and characterization of bactericidal wound dressing material based on gelatin nanofiber. Int. J. Biol. Macromol..

[40] Reshmi C., Suja P., Manaf O., Sanu P., Sujith A. (2018). Nanochitosan enriched poly ε-caprolactone electrospun wound dressing membranes: A fine tuning of physicochemical properties, hemocompatibility and curcumin release profile. Int. J. Biol. Macromol..

[41] Xie Y., Yi Z.-X., Wang J.-X., Hou T.-G., Jiang Q. (2018). Carboxymethyl konjac glucomannan-crosslinked chitosan sponges for wound dressing. Int. J. Biol. Macromol..

[42] Tao G., Wang Y., Cai R., Chang H., Song K., Zuo H., Zhao P., Xia Q., He H. (2019). Design and performance of sericin/poly (vinyl alcohol) hydrogel as a drug delivery carrier for potential wound dressing application. Mater. Sci. Eng. C.

[43] Hadisi Z., Nourmohammadi J., Nassiri S.M. (2018). The antibacterial and anti-inflammatory investigation of Lawsonia Inermis-gelatin-starch nano-fibrous dressing in burn wound. Int. J. Biol. Macromol..

[44] Tamahkar E., Özkahraman B., Süloğlu A.K., İdil N., Perçin I. (2020). A novel multilayer hydrogel wound dressing for antibiotic release. J. Drug Deliv. Sci. Technol..

[45] Rodrigues M., Kosaric N., Bonham C.A., Gurtner G.C. (2019). Wound healing: A cellular perspective. Physiol. Rev..

[46] Bechstein W.O. (2018). Towards simpler and reliable wound care. Dtsch. Ärztebl. Int..

[47] Li X., Wang C., Yang S., Liu P., Zhang B. (2018). Electrospun Pcl/mupirocin and chitosan/lidocaine hydrochloride multifunctional double layer nanofibrous scaffolds for wound dressing applications. Int. J. Nanomed..

[48] Agrawal A., Purwar R. (2018). Swelling and drug release kinetics of composite wound dressing. Indian J. Fibre Text. Res..

[49] Noori S., Kokabi M., Hassan Z. (2018). Poly (vinyl alcohol)/chitosan/honey/clay responsive nanocomposite hydrogel wound dressing. J. Appl. Polym. Sci..

[50] Zou P., Lee W.-H., Gao Z., Qin D., Wang Y., Liu J., Sun T., Gao Y. (2020). Wound dressing from polyvinyl alcohol/chitosan electrospun fiber membrane loaded with OH-CATH30 nanoparticles. Carbohydr. Polym..

[51] Unnithan A.R., Ghavami Nejad A., Sasikala A.R.K., Thomas R.G., Jeong Y.Y., Murugesan P., Nasseri S., Wu D., Park C.H., Kim C.S. (2016). Electrospun zwitterionic nanofibers with in situ decelerated epithelialization property for non-adherent and easy removable wound dressing application. Chem. Eng. J..

[52] Zhou B., Li Y., Deng H., Hu Y., Li B. (2014). Antibacterial multilayer films fabricated by layer-by-layer immobilizing lysozyme and gold nanoparticles on nanofibers. Colloids Surf. B Biointerfaces.

[53] Chen H., Lan G., Ran L., Xiao Y., Yu K., Lu B., Dai F., Wu D., Lu F. (2018). A novel wound dressing based on a Konjac glucomannan/silver nanoparticle composite sponge effectively kills bacteria and accelerates wound healing. Carbohydr. Polym..

[54] Feng Y., Li X., Zhang Q., Yan S., Guo Y., Li M., You R. (2019). Mechanically robust and flexible silk protein/polysaccharide composite sponges for wound dressing. Carbohydr. Polym..

[55] Ma R., Wang Y., Qi H., Shi C., Wei G., Xiao L., Huang Z., Liu S., Yu H., Teng C. (2019). Nanocomposite sponges of sodium alginate/graphene oxide/polyvinyl alcohol as potential wound dressing: In vitro and in vivo evaluation. Compos. Part B Eng..

[56] Axibal E., Brown M. (2019). Surgical dressings and novel skin substitutes. Dermatol. Clin..

[57] Sayed M., Nouh O., Ahmed A., Gaber Anany M., El Rachidi N., Salem A. (2019). A randomized control trial comparing transparent film dressings and conventional occlusive dressings for elective surgical procedures. Open Access Maced. J. Med. Sci..

[58] Weller C., Team V., Rajendran S. (2019). Interactive dressings and their role in moist wound management. Advanced Textiles for Wound Care (Second Edition).

[59] Aderibigbe B.A., Buyana B. (2018). Alginate in wound dressings. Pharmaceutics.

[60] Tate S., Price A., Harding K. (2018). Dressings for venous leg ulcers. BMJ.

[61] Dhivya S., Padma V.V., Santhini E. (2015). Wound dressings—A review. BioMedicine.

[62] Banks V., Bale S., Harding K., Harding E.F. (1997). Evaluation of a new polyurethane foam dressing. J. Wound Care.

[63] Maver T., Maver U., Pivec T., Kurečič M., Persin Z., Kleinschek K.S. (2018). Advanced wound care. Bioactive Polysaccharide Materials for Modern Wound Healing.

[64] Song E.-H., Jeong S.-H., Park J.-U., Kim S., Kim H.-E., Song J. (2017). Polyurethane-silica hybrid foams from a one-step foaming reaction, coupled with a sol-gel process, for enhanced wound healing. Mater. Sci. Eng. C.

[65] Erring M., Gaba S., Mohsina S., Tripathy S., Sharma R.K. (2019). Comparison of efficacy of silver-nanoparticle gel, nano-silver-foam and collagen dressings in treatment of partial thickness burn wounds. Burns.

[66] Baljit S., Rajneesh, Baldev S., Kumar A., Aery S. (2019). Polysaccharides sterculia gum/psyllium based hydrogel dressings for drug delivery applications. Polym. Sci. Ser. A.

[67] Pan H., Fan D., Duan Z., Zhu C., Fu R., Li X. (2019). Non-stick hemostasis hydrogels as dressings with bacterial barrier activity for cutaneous wound healing. Mater. Sci. Eng. C.

[68] Rajendran N.K., Kumar S.S.D., Houreld N.N., Abrahamse H. (2018). A review on nanoparticle based treatment for wound healing. J. Drug Deliv. Sci. Technol..

[69] Dhaliwal K., Lopez N. (2018). Hydrogel dressings and their application in burn wound care. Br. J. Commun. Nurs..

[70] Qu J., Zhao X., Liang Y., Xu Y., Ma P.X., Guo B. (2019). Degradable conductive injectable hydrogels as novel antibacterial, anti-oxidant wound dressings for wound healing. Chem. Eng. J..

[71] Martin F.T., O’Sullivan J.B., Regan P.J., McCann J., Kelly J.L. (2010). Hydrocolloid dressing in pediatric burns may decrease operative intervention rates. J. Pediatr. Surg..

[72] Zhang L., Yin H., Lei X., Lau J.N.Y., Yuan M., Wang X., Zhang F., Zhou F., Qi S., Shu B. (2019). A systematic review and meta-analysis of clinical effectiveness and safety of hydrogel dressings in the management of skin wounds. Front. Bioeng. Biotechnol..

[73] Pannerselvam B., Dharmalingam Jothinathan M.K., Rajenderan M., Perumal P., Pudupalayam Thangavelu K., Kim H.J., Singh V., Rangarajulu S.K. (2017). An in vitro study on the burn wound healing activity of cotton fabrics incorporated with phytosynthesized silver nanoparticles in male Wistar albino rats. Eur. J. Pharm. Sci..

[74] Ullah F., Javed F., Khan A.N., Kudus M.H.A., Jamila N., Minhaz A., Akil H.M. (2019). Synthesis and surface modification of chitosan built nanohydrogel with antiviral and antimicrobial agent for controlled drug delivery. Biointerface Res. Appl. Chem..

[75] Madaghiele M., Demitri C., Sannino A., Ambrosio L. (2014). Polymeric hydrogels for burn wound care: Advanced skin wound dressings and regenerative templates. Burns Trauma.

[76] Shi C., Wang C., Liu H., Li Q., Li R., Zhang Y., Liu Y., Shao Y., Wang J. (2020). Selection of Appropriate Wound Dressing for Various Wounds. Front Bioeng Biotechnol..

[77] Ullah F., Javed F., Zakaria M.R., Jamila N., Khattak R., Khan A.N., Akil H.M. (2019). Determining the molecular-weight and interfacial properties of chitosan built nanohydrogel for controlled drug delivery applications. Biointerface Res. Appl. Chem..

[78] Francesko A., Petkova P., Tzanov T. (2018). Hydrogel dressings for advanced wound management. Curr. Med. Chem..

[79] Hussain Z., Thu H.E., Shuid A.N., Katas H., Hussain F. (2018). Recent advances in polymer-based wound dressings for the treatment of diabetic foot ulcer: An overview of state-of-the-art. Curr. Drug Targ..

[80] Gupta A., Kowalczuk M., Heaselgrave W., Britland S.T., Martin C., Radecka I. (2019). The production and application of hydrogels for wound management: A review. Eur. Polym. J..

[81] Naseri-Nosar M., Ziora Z.M. (2018). Wound dressings from naturally-occurring polymers: A review on homopolysaccharide-based composites. Carbohydr. Polym..

[82] Mir M., Ali M.N., Barakullah A., Gulzar A., Arshad M., Fatima S., Asad M. (2018). Synthetic polymeric biomaterials for wound healing: A review. Prog. Biomater..

[83] Stubbe B., Mignon A., Declercq H., Van Vlierberghe S., Dubruel P. (2019). Development of gelatin-alginate hydrogels for burn wound treatment. Macromol. Biosci..

[84] Nuutila K., Grolman J., Yang L., Broomhead M., Lipsitz S., Onderdonk A., Mooney D., Eriksson E. (2020). Immediate treatment of burn wounds with high concentrations of topical antibiotics in an alginate hydrogel using a platform wound device. Adv. Wound Care.

[85] Abdelghany A.M., Meikhail M.S., El-Bana A.A. (2019). Microbial activity and swelling behavior of chitosan/polyvinyl alcohol/sodium alginate semi-natural terpolymer interface containing amoxicillin for wound dressing applications. Biointerface Res. Appl. Chem..

[86] Wang T., Zhu X.-K., Xue X.-T., Wu D.-Y. (2012). Hydrogel sheets of chitosan, honey and gelatin as burn wound dressings. Carbohydr. Polym..

[87] Hamedi H., Moradi S., Hudson S.M., Tonelli A.E. (2018). Chitosan based hydrogels and their applications for drug delivery in wound dressings: A review. Carbohydr. Polym..

[88] Zhai M., Xu Y., Zhou B., Jing W. (2018). Keratin-chitosan/n-ZnO nanocomposite hydrogel for antimicrobial treatment of burn wound healing: Characterization and biomedical application. J. Photochem. Photobiol. B Biol..

[89] Oryan A., Jalili M., Kamali A., Nikahval B. (2018). The concurrent use of probiotic microorganism and collagen hydrogel/scaffold enhances burn wound healing: An in vivo evaluation. Burns.

[90] Ge B., Wang H., Li J., Liu H., Yin Y., Zhang N., Qin S. (2020). Comprehensive assessment of nile tilapia skin (oreochromis niloticus) collagen hydrogels for wound dressings. Mar. Drugs.

[91] Gharibi R., Kazemi S., Yeganeh H., Tafakori V. (2019). Utilizing dextran to improve hemocompatibility of antimicrobial wound dressings with embedded quaternary ammonium salts. Int. J. Biol. Macromol..

[92] Zhu Q., Jiang M., Liu Q., Yan S., Feng L., Lan Y., Shan G., Xue W., Guo R. (2018). Enhanced healing activity of burn wound infection by a dextran-HA hydrogel enriched with sanguinarine. Biomater. Sci..

[93] Li Z., Zhou F., Li Z., Lin S., Chen L., Liu L., Chen Y. (2018). Hydrogel cross-linked with dynamic covalent bonding and micellization for promoting burn wound healing. ACS Appl. Mater. Interfaces.

[94] Shawan M.M.A.K., Islam N., Aziz S., Khatun N., Sarker S.R., Hossain M., Hossan T., Morshed M., Sarkar M., Shakil M.S. (2019). Fabrication of xanthan gum: Gelatin (Xnt: Gel) hybrid composite hydrogels for evaluating skin wound healing efficacy. Mod. Appl. Sci..

[95] Alves A., Miguel P.S., Araujo R.T.S.A., de Jesús Valle J.M., Sánchez Navarro A., Correia J.I., Ribeiro P.M., Coutinho P. (2020). Xanthan gum–Konjac glucomannan blend hydrogel for wound healing. Polymers.

[96] Naghibzadeh M., Firoozi S., Nodoushan F.S., Adabi M., Khoradmehr A., Fesahat F., Esnaashari S.S., Khosravani M., Adabi M., Tavakol S. (2018). application of electrospun gelatin nanofibers in tissue engineering. Biointerface Res. Appl. Chem..

[97] Hunter A.C., Moghimi S.M. (2002). Therapeutic synthetic polymers: A game of Russian roulette?. Drug Discov. Today.

[98] Mohandas A., Kumar P.T.S., Raja B., Lakshmanan V.-K., Jayakumar R. (2015). Exploration of alginate hydrogel/nano zinc oxide composite bandages for infected wounds. Int. J. Nanomed..

[99] De Cicco F., Reverchon E., Adami R., Auriemma G., Russo P., Calabrese E.C., Porta A., Aquino R.P., Del Gaudio P. (2014). In situ forming antibacterial dextran blend hydrogel for wound dressing: SAA technology vs. spray drying. Carbohydr. Polym..

[100] Suvarnapathaki S., Nguyen M.A., Wu X., Nukavarapu S.P., Camci-Unal G. (2019). Synthesis and characterization of photocrosslinkable hydrogels from bovine skin gelatin. RSC Adv..

[101] Huang W., Wang Y., Huang Z., Wang X., Chen L., Zhang Y., Zhang L. (2018). On-demand dissolvable self-healing hydrogel based on carboxymethyl chitosan and cellulose nanocrystal for deep partial thickness burn wound healing. ACS Appl. Mater. Interfaces.

[102] Ying H., Zhou J., Wang M., Su D., Ma Q., Lv G., Chen J. (2019). In situ formed collagen-hyaluronic acid hydrogel as biomimetic dressing for promoting spontaneous wound healing. Mater. Sci. Eng. C.

[103] Sun G., Zhang X., Shen Y.-I., Sebastian R., Dickinson L.E., Fox-Talbot K., Reinblatt M., Steenbergen C., Harmon J.W., Gerecht S. (2011). Dextran hydrogel scaffolds enhance angiogenic responses and promote complete skin regeneration during burn wound healing. Proc. Natl. Acad. Sci. USA.

[104] Lin S.-P., Kung H.-N., Tsai Y.-S., Tseng T.-N., Hsu K.-D., Cheng K.-C. (2017). Novel dextran modified bacterial cellulose hydrogel accelerating cutaneous wound healing. Cellulose.

[105] Li X., Li A., Feng F., Jiang Q., Sun H., Chai Y., Yang R., Wang Z., Hou J., Li R. (2019). Effect of the hyaluronic acid-poloxamer hydrogel on skin-wound healing: In vitro and in vivo studies. Anim. Model Exp. Med..

[106] Zhu J., Li F., Wang X., Yu J., Wu D. (2018). Hyaluronic acid and polyethylene glycol hybrid hydrogel encapsulating nanogel with hemostasis and sustainable antibacterial property for wound healing. ACS Appl. Mater. Interfaces.

[107] Singh S., Gupta A., Sharma D., Gupta B. (2018). Dextran based herbal nanobiocomposite membranes for scar free wound healing. Int. J. Biol. Macromol..

[108] Zheng C., Liu C., Chen H., Wang N., Liu X., Sun G., Qiao W. (2019). Effective wound dressing based on Poly (vinyl alcohol)/Dextran-aldehyde composite hydrogel. Int. J. Biol. Macromol..

[109] Graça M.F., Miguel S.P., Cabral C.S., Correia I.J. (2020). Hyaluronic acid-based wound dressings: A review. Carbohydr. Polym..

[110] Abou-Okeil A., Fahmy H., El-Bisi M., Ahmed-Farid O. (2018). Hyaluronic acid/Na-alginate films as topical bioactive wound dressings. Eur. Polym. J..

[111] Lin Z., Wu T., Wang W., Li B., Wang M., Chen L., Xia H., Zhang T. (2019). Biofunctions of antimicrobial peptide-conjugated alginate/hyaluronic acid/collagen wound dressings promote wound healing of a mixed-bacteria-infected wound. Int. J. Biol. Macromol..

[112] Singhvi G., Hans N., Shiva N., Dubey S.K., Hasnain M.S., Nayak A.K. (2019). Xanthan gum in drug delivery applications. Natural Polysaccharides in Drug Delivery and Biomedical Applications.

[113] Kumar A., Rao K.M., Han S.S. (2018). Application of xanthan gum as polysaccharide in tissue engineering: A review. Carbohydr. Polym..

[114] Raafat A.I., El-Sawy N.M., Badawy N.A., Mousa E.A., Mohamed A.M. (2018). Radiation fabrication of Xanthan-based wound dressing hydrogels embedded ZnO nanoparticles: In vitro evaluation. Int. J. Biol. Macromol..

[115] Joshi Navare K., Eggermont L.J., Rogers Z.J., Mohammed H.S., Colombani T., Bencherif S.A., Li B., Moriarty T.F., Webster T., Xing M. (2020). Antimicrobial Hydrogels: Key Considerations and Engineering Strategies for Biomedical Applications. Racing for the Surface: Pathogenesis of Implant Infection and Advanced Antimicrobial Strategies.

[116] Yang K., Han Q., Chen B., Zheng Y., Zhang K., Li Q., Wang J. (2018). Antimicrobial hydrogels: Promising materials for medical application. Int. J. Nanomed..

[117] Simões D., Miguel S.P., Ribeiro M.P., Coutinho P., Mendonça A.G., Correia I.J. (2018). Recent advances on antimicrobial wound dressing: A review. Eur. J. Pharm. Biopharm..

[118] Gupta A., Briffa S.M., Swingler S., Gibson H., Kannappan V., Adamus G., Kowalczuk M., Martin C., Radecka I. (2020). Synthesis of silver nanoparticles using curcumin-cyclodextrins loaded into bacterial cellulose-based hydrogels for wound dressing applications. Biomacromolecules.

[119] Liu H., Wang C., Li C., Qin Y., Wang Z., Yang F., Li Z., Wang J. (2018). A functional chitosan-based hydrogel as a wound dressing and drug delivery system in the treatment of wound healing. RSC Adv..

[120] Boonkaew B., Kempf M., Kimble R., Supaphol P., Cuttle L. (2014). Antimicrobial efficacy of a novel silver hydrogel dressing compared to two common silver burn wound dressings: Acticoat™ and PolyMem Silver^®^. Burns.

[121] Kim M.H., Park H., Nam H.C., Park S.R., Jung J.-Y., Park W.H. (2018). Injectable methylcellulose hydrogel containing silver oxide nanoparticles for burn wound healing. Carbohydr. Polym..

[122] Banerjee J., Seetharaman S., Wrice N.L., Christy R.J., Natesan S. (2019). Delivery of silver sulfadiazine and adipose derived stem cells using fibrin hydrogel improves infected burn wound regeneration. PLoS ONE.

[123] Mohandas A., Deepthi S., Biswas R., Jayakumar R. (2018). Chitosan based metallic nanocomposite scaffolds as antimicrobial wound dressings. Bioact. Mater..

[124] Li Z., Cheng J., Yang X., Liu H., Xu X., Ma L., Shang S., Song Z. (2020). Construction of antimicrobial and biocompatible cotton textile based on quaternary ammonium salt from rosin acid. Int. J. Biol. Macromol..

[125] Zhou D., Yang R., Yang T., Xing M., Luo G. (2018). Preparation of chitin-amphipathic anion/quaternary ammonium salt ecofriendly dressing and its effect on wound healing in mice. Int. J. Nanomed..

[126] Nimal T., Baranwal G., Bavya M., Biswas R., Jayakumar R. (2016). Anti-staphylococcal activity of injectable nano tigecycline/chitosan-PRP composite hydrogel using drosophila melanogaster model for infectious wounds. ACS Appl. Mater. Interfaces.

[127] Nešović K., Janković A., Radetić T., Vukašinović-Sekulić M., Kojić V., Živković L., Perić-Grujić A., Rhee K.Y., Mišković-Stanković V. (2019). Chitosan-based hydrogel wound dressings with electrochemically incorporated silver nanoparticles—In vitro study. Eur. Polym. J..

[128] Nešović K., Janković A., Kojić V., Vukašinović-Sekulić M., Perić-Grujić A., Rhee K.Y., Mišković-Stanković V. (2018). Silver/poly(vinyl alcohol)/chitosan/graphene hydrogels—Synthesis, biological and physicochemical properties and silver release kinetics. Compos. Part B Eng..

[129] Sabry N.M., Tolba S., Abdel-Gawad F.K., Bassem S.M., Nassar H.F., El-Taweel G.E., Okasha A., Ibrahim M. (2018). Interaction between nano silver and bacteria: Modeling approach. Biointerface Res. Appl. Chem..

[130] Xie Y., Liao X., Zhang J., Yang F., Fan Z. (2018). Novel chitosan hydrogels reinforced by silver nanoparticles with ultrahigh mechanical and high antibacterial properties for accelerating wound healing. Int. J. Biol. Macromol..

[131] Boateng J., Catanzano O. (2020). Silver and silver nanoparticle-based antimicrobial dressings. Ther. Dress. Wound Heal Appl..

[132] Vuković J.S., Perić-Grujić A.A., Mitić-Ćulafić D.S., Božić Nedeljković B.D., Tomić S.L. (2020). Antibacterial activity of pH-sensitive silver(I)/poly(2-hydroxyethyl acrylate/itaconic acid) hydrogels. Macromol. Res..

[133] Wang Y.-L., Zhou Y.-N., Li X.-Y., Huang J., Wahid F., Zhong C., Chu L.-Q. (2020). Continuous production of antibacterial carboxymethyl chitosan-zinc supramolecular hydrogel fiber using a double-syringe injection device. Int. J. Biol. Macromol..

[134] Sabry N.M., Tolba S.T.M., Abdel-Gawad F.K., Bassem S.M., Nassar H., El-Taweel G.E., Ibrahim M.A. (2018). On the molecular modeling analyses of the interaction between nano zinc oxide and bacteria. Biointerface Res. Appl. Chem..

[135] Khorasani M.T., Joorabloo A., Moghaddam A., Shamsi H., MansooriMoghadam Z. (2018). Incorporation of ZnO nanoparticles into heparinised polyvinyl alcohol/chitosan hydrogels for wound dressing application. Int. J. Biol. Macromol..

[136] Rakhshaei R., Namazi H. (2017). A potential bioactive wound dressing based on carboxymethyl cellulose/ZnO impregnated MCM-41 nanocomposite hydrogel. Mater. Sci. Eng. C.

[137] Khorasani M.T., Joorabloo A., Adeli H., Mansoori-Moghadam Z., Moghaddam A. (2019). Design and optimization of process parameters of polyvinyl (alcohol)/chitosan/nano zinc oxide hydrogels as wound healing materials. Carbohydr. Polym..

[138] Mateus D., Marto J., Trindade P., Gonçalves H., Salgado A., Machado P., Melo-Gouveia A., Ribeiro M.H., Almeida J.A. (2019). Improved morphine-loaded hydrogels for wound-related pain relief. Pharmaceutics.

[139] Aycan D., Selmi B., Kelel E., Yildirim T., Alemdar N. (2019). Conductive polymeric film loaded with ibuprofen as a wound dressing material. Eur. Polym. J..

[140] Eroglu I., Gultekinoglu M., Bayram C., Erikci A., Ciftci S.Y., Ayse Aksoy E., Ulubayram K. (2019). Gel network comprising UV crosslinked PLGA-b-PEG-MA nanoparticles for ibuprofen topical delivery. Pharm. Dev. Technol..

[141] Sanchez M.F., Breda S.A., Soria E.A., Tártara L.I., Manzo R.H., Olivera M.E. (2018). Ciprofloxacin-lidocaine-based hydrogel: Development, characterization, and in vivo evaluation in a second-degree burn model. Drug Deliv. Transl. Res..

[142] Yaşayan G., Karaca G., Akgüner Z.P., Bal Öztürk A. (2020). Chitosan/collagen composite films as wound dressings encapsulating allantoin and lidocaine hydrochloride. Int. J. Polym. Mater. Polym. Biomater..

[143] ter Horst B., Chouhan G., Moiemen N.S., Grover L.M. (2018). Advances in keratinocyte delivery in burn wound care. Adv. Drug Deliv. Rev..

[144] Wang P., Huang S., Hu Z., Yang W., Lan Y., Zhu J., Hancharou A., Guo R., Tang B. (2019). In situ formed anti-inflammatory hydrogel loading plasmid DNA encoding VEGF for burn wound healing. Acta Biomater..

[145] Catanzano O., Boateng J., Boateng J. (2020). Local Delivery of Growth Factors Using Wound Dressings. Therapeutic Dressings and Wound Healing Applications.

[146] Oryan A., Alemzadeh E., Mohammadi A.A., Moshiri A. (2019). Healing potential of injectable Aloe vera hydrogel loaded by adipose-derived stem cell in skin tissue-engineering in a rat burn wound model. Cell Tissue Res..

[147] Zhou S., Hokugo A., McClendon M., Zhang Z., Bakshi R., Wang L., Segovia L.A., Rezzadeh K., Stupp S.I., Jarrahy R. (2019). Bioactive peptide amphiphile nanofiber gels enhance burn wound healing. Burns.

[148] Khan A., Xu M., Wang T., You C., Wang X., Ren H., Zhou H., Khan A., Han C., Li P. (2019). Catechol cross-linked antimicrobial peptide hydrogels prevent multidrug-resistant *Acinetobacter baumannii* infection in burn wounds. Biosci. Rep..

[149] Wang W., Liu G., Liu M., Li X. (2020). Mechanisms underlying the action of self-assembling short-peptide nano-fiber gel scaffold materials in the aesthetic repair of burn wounds. Mater. Express.

[150] Djekic L., Martinović M., Ćirić A., Fraj J. (2020). Composite chitosan hydrogels as advanced wound dressings with sustained ibuprofen release and suitable application characteristics. Pharm. Dev. Technol..

[151] Jiji S., Udhayakumar S., Rose C., Muralidharan C., Kadirvelu K. (2019). Thymol enriched bacterial cellulose hydrogel as effective material for third degree burn wound repair. Int. J. Biol. Macromol..

[152] Dang L.H., Huynh N.T., Pham N.O., Nguyen C.T., Vu M.T., Dinh V.T., Le V.T., Tran N.Q. (2019). Injectable nanocurcumin-dispersed gelatin–pluronic nanocomposite hydrogel platform for burn wound treatment. Bull. Mater. Sci..

[153] Comotto M., Saghazadeh S., Bagherifard S., Aliakbarian B., Kazemzadeh-Narbat M., Sharifi F., Mousavi Shaegh S.A., Arab-Tehrany E., Annabi N., Perego P. (2019). Breathable hydrogel dressings containing natural antioxidants for management of skin disorders. J. Biomater. Appl..

[154] Ehterami A., Salehi M., Farzamfar S., Samadian H., Vaez A., Sahrapeyma H., Ghorbani S. (2020). A promising wound dressing based on alginate hydrogels containing vitamin D3 cross-linked by calcium carbonate/d-glucono-δ-lactone. Biomed. Eng. Lett..

[155] Ehterami A., Salehi M., Farzamfar S., Samadian H., Vaez A., Ghorbani S., Ai J., Sahrapeyma H. (2019). Chitosan/alginate hydrogels containing Alpha-tocopherol for wound healing in rat model. J. Drug. Deliv. Sci. Technol..

[156] Yin F., Lin L., Zhan S. (2019). Preparation and properties of cellulose nanocrystals, gelatin, hyaluronic acid composite hydrogel as wound dressing. J. Biomater. Sci. Polym. Ed..

[157] Selvakumar G., Iyappan K., Suguna L. (2020). Biomaterials and wound healing: A mini review. Ann. Oper. Surg..

[158] Mohamad N., Loh E.Y.X., Fauzi M.B., Ng M.H., Mohd Amin M.C.I. (2019). In vivo evaluation of bacterial cellulose/acrylic acid wound dressing hydrogel containing keratinocytes and fibroblasts for burn wounds. Drug Deliv. Transl. Res..

[159] Ternullo S., Schulte Werning L.V., Holsæter A.M., Škalko-Basnet N. (2020). Curcumin-in-deformable liposomes-in-chitosan-hydrogel as a novel wound dressing. Pharmaceutics.

[160] Wu Y.C., Wu G.X., Huang H.H., Kuo S.M. (2019). Liposome-encapsulated farnesol accelerated tissue repair in third-degree burns on a rat model. Burns.

[161] Pilehvar-Soltanahmadi Y., Dadashpour M., Mohajeri A., Fattahi A., Sheervalilou R., Zarghami N. (2018). An overview on application of natural substances incorporated with electrospun nanofibrous scaffolds to development of innovative wound dressings. Mini Rev. Med. Chem..

[162] Soto-Chilaca G.A., Mejía-Garibay B., Navarro-Amador R., Ramírez-Corona N., Palou E., López-Malo A. (2019). Cinnamaldehyde-loaded chitosan nanoparticles: Characterization and antimicrobial activity. Biointerface Res. Appl. Chem..

[163] Bahramian G., Golestan L., Khosravi-Darani K. (2018). Antimicrobial and antioxidant effect of nanoliposomes containing zataria multiflora boiss essential oil on the rainbow trout fillets during refrigeration. Biointerface Res. Appl. Chem..

[164] Dos Santos L.D.R., Dos Santos A.E.S., Cerávolo I.P., Figueiredo F.J.B., Dias-Souza M.V. (2018). Antibiofilm activity of black tea leaf extract, its cytotoxicity and interference on the activity of antimicrobial drugs. Biointerface Res. Appl. Chem..

[165] Dias-Souza M.V., Dias C.G., Ferreira-Marçal P.H. (2018). Interactions of natural products and antimicrobial drugs: Investigations of a dark matter in chemistry. Biointerface Res. Appl. Chem..

[166] Veerasubramanian P.K., Thangavel P., Kannan R., Chakraborty S., Ramachandran B., Suguna L., Muthuvijayan V. (2018). An investigation of konjac glucomannan-keratin hydrogel scaffold loaded with Avena sativa extracts for diabetic wound healing. Colloids Surf. B Biointerfaces.

[167] Neto R.J.G., Genevro G.M., de Almeida Paulo L., Lopes P.S., de Moraes M.A., Beppu M.M. (2019). Characterization and in vitro evaluation of chitosan/konjac glucomannan bilayer film as a wound dressing. Carbohydr. Polym..

[168] Zhou L., Xu T., Yan J., Li X., Xie Y., Chen H. (2020). Fabrication and characterization of matrine-loaded konjac glucomannan/fish gelatin composite hydrogel as antimicrobial wound dressing. Food Hydrocoll..

[169] Mirzaei B., Etemadian S., Goli H.R., Bahonar S., Gholami S.A., Karami P., Farhadi M., Tavakoli R. (2018). Construction and analysis of alginate-based honey hydrogel as an ointment to heal of rat burn wound related infections. Int. J. Burns Trauma.

[170] Febriyenti F., Lucida H., Almahdy A., Alfikriyah I., Hanif M. (2019). Wound-healing effect of honey gel and film. J. Pharm. Bioallied Sci..

[171] Fathollahipour S., Koosha M., Tavakoli J., Maziarfar S., Fallah Mehrabadi J. (2020). Erythromycin releasing PVA/sucrose and PVA/honey hydrogels as wound dressings with antibacterial activity and enhanced bio-adhesion. Iran. J. Pharm. Res..

[172] Mohd Zohdi R., Abu Bakar Zakaria Z., Yusof N., Mohamed Mustapha N., Abdullah M.N.H. (2012). Gelam (*Melaleuca* spp.) honey-based hydrogel as burn wound dressing. Evid. Based Complement. Alternat. Med..

[173] Loh E.Y.X., Mohamad N., Fauzi M.B., Ng M.H., Ng S.F., Amin M.C.I.M. (2018). Development of a bacterial cellulose-based hydrogel cell carrier containing keratinocytes and fibroblasts for full-thickness wound healing. Sci. Rep..

[174] Rodriguez-Chanfrau J.E., Veranes-Pantoja Y., Basmaji P., Guastaldi A.C. (2019). Influence of the reaction time during the treatment of bacterial cellulose with sulfuric acid solution. Biointerface Res. Appl. Chem..

[175] Poletti S., Lucke L.D., Acunha R., Mattos M., Gaspi F. (2018). Electromagnetic stimulation combined with aloe vera increases collagen reorganization in burn repair. J. Pharm. Pharmacol..

[176] Singh S., Gupta A., Gupta B. (2018). Scar free healing mediated by the release of aloe vera and manuka honey from dextran bionanocomposite wound dressings. Int. J. Biol. Macromol..

[177] Yates K.M., Proctor C.A., Atchley D.H. (2012). Antimicrobial Silver Hydrogel Composition for the Treatment of Burns and Wounds.

[178] Rahman M.S., Islam R., Rana M.M., Spitzhorn L.-S., Rahman M.S., Adjaye J., Asaduzzaman S.M. (2019). Characterization of burn wound healing gel prepared from human amniotic membrane and Aloe vera extract. BMC Complement. Alternat. Med..

[179] Murphy S.V., Skardal A., Atala A. (2018). Amniotic Membrane Hydrogel and Methods of Making.

[180] Hossain M.L., Rahman M.A., Siddika A., Adnan M., Rahman H., Diba F., Hasan M.Z., Asaduzzaman S. (2019). Burn and wound healing using radiation sterilized human amniotic membrane and centella asiatica derived gel: A review. Regen. Eng. Transl. Med..

[181] Rana M.M., Rahman M.S., Ullah M.A., Siddika A., Hossain M.L., Akhter M.S., Hasan M.Z., Asaduzzaman S.M. (2020). Amnion and collagen-based blended hydrogel improves burn healing efficacy on rat skin wound model in presence of wound dressing biomembrane. BioMed. Mater. Eng..

[182] Murphy S.V., Skardal A., Nelson Jr R.A., Sunnon K., Reid T., Clouse C., Kock N.D., Jackson J., Soker S., Atala A. (2020). Amnion membrane hydrogel and amnion membrane powder accelerate wound healing in a full thickness porcine skin wound model. Stem Cells Transl. Med..

[183] Farhadihosseinabadi B., Farahani M., Tayebi T., Jafari A., Biniazan F., Modaresifar K., Moravvej H., Bahrami S., Redl H., Tayebi L. (2018). Amniotic membrane and its epithelial and mesenchymal stem cells as an appropriate source for skin tissue engineering and regenerative medicine. Artif. Cells Nanomed. Biotechnol..

[184] Hossain M., Islam M., Diba F., Hasan M., Asaduzzaman S. (2018). The synergistic effect of AM and MO derived gel in burn and wound healing. Int. J. Complement. Alt. Med..

